# Cytoplasmic DNAs: Sources, sensing, and roles in the development of lung inflammatory diseases and cancer

**DOI:** 10.3389/fimmu.2023.1117760

**Published:** 2023-04-12

**Authors:** Xintong He, Ye Sun, Jianzhang Lu, Faiza Naz, Shenglin Ma, Jian Liu

**Affiliations:** ^1^ Department of Respiratory and Critical Care Medicine, The Second Affiliated Hospital, Zhejiang University School of Medicine, Zhejiang University, Hangzhou, China; ^2^ Zhejiang University-University of Edinburgh Institute (ZJU-UoE Institute), Zhejiang University School of Medicine, Zhejiang University, Haining, China; ^3^ College of Medicine and Veterinary Medicine, The University of Edinburgh, Edinburgh, United Kingdom; ^4^ Hangzhou Cancer Institution, Affiliated Hangzhou Cancer Hospital, Zhejiang University School of Medicine, Zhejiang University, Hangzhou, China; ^5^ Cancer Center, Zhejiang University, Hangzhou, China; ^6^ Biomedical and Heath Translational Research Center of Zhejiang Province, Haining, China; ^7^ Dr. Li Dak Sum & Yip Yio Chin Center for Stem Cell and Regenerative Medicine, Zhejiang University, Hangzhou, China

**Keywords:** cytoplasmic DNA, innate immunity, interferon, immunotherapy, COVID-19, cytokine, STING, lung cancer

## Abstract

Cytoplasmic DNA is emerging as a pivotal contributor to the pathogenesis of inflammatory diseases and cancer, such as COVID-19 and lung carcinoma. However, the complexity of various cytoplasmic DNA-related pathways and their crosstalk remains challenging to distinguish their specific roles in many distinct inflammatory diseases, especially for the underlying mechanisms. Here, we reviewed the latest findings on cytoplasmic DNA and its signaling pathways in inflammatory lung conditions and lung cancer progression. We found that sustained activation of cytoplasmic DNA sensing pathways contributes to the development of common lung diseases, which may result from external factors or mutations of key genes in the organism. We further discussed the interplays between cytoplasmic DNA and anti-inflammatory or anti-tumor effects for potential immunotherapy. In sum, this review aids in understanding the roles of cytoplasmic DNAs and exploring more therapeutic strategies.

## 1 Introduction

Lung diseases have long been regarded as the leading cause of death. Acute lung inflammation, a type of lung disease with relatively mild symptoms caused by acute respiratory infection, will gradually develop into chronic types if not treated thoroughly, which even further leads to lung cancers (1–4). Data shows that lower respiratory infections such as pneumonia afflict about 489 million people each year (1). In the current coronavirus disease 2019 (COVID-19) pandemic, hundreds of millions of people have been infected with severe acute respiratory syndrome coronavirus 2 (SARS-CoV-2), among which over 2 million people lost their lives (1, 5). Furthermore, as the most common and severe kind of chronic disease, lung cancer kills over 2 million people annually (6). Therefore, the treatment of lung diseases is essential for the safeguarding of human health.

The corresponding treatment for pneumonia escalates from the traditional antibiotic treatment to more abundant means with lung ultrasound and molecular biology diagnoses and DNA-based therapeutic approaches, such as the delivery of anti-TNF-siRNA (7, 8). As for lung cancer, the research and treatments are more varied. Current researches focus on the mechanism at the molecule level, involving gene mutations, the complexity of the tumor microenvironment, and the change of epigenetics (9–11). In addition, treatments including chemotherapy, radiotherapy, surgical resection, immune therapy such as CAR-T and CAR-NK cells, and target therapy such as epigenetics targeted drugs and epidermal growth factor receptor inhibitors have been widely applied (9, 11–13). However, current therapies are limited to specific types of lung diseases and have fewer effects on elderly people (1, 9, 14). Therefore, new therapeutic strategies are required.

Recent studies have established the essential role of cytoplasmic DNA in acute and chronic lung inflammatory diseases and lung cancer (15, 16). DNA stores biological information whose regular locations (*e.g.*, nuclei or mitochondria) are important for cell function and stability. Its mislocalization in areas free of DNA, such as cytoplasm, will be sensed and trigger the cell responses, a part of innate immune systems (15). In innate immunity, the host provides the first defense line by developing pattern-recognition receptors (PRRs) to recognize pathogen-associated molecular patterns (PAMPs) or damage-associated molecular patterns (DAMPs) derived. DNA released from invading pathogens to host cell cytoplasm is a kind of PAMP, and self-DNA damaged by cell stresses abnormally transferred to the cytoplasm is a kind of DAMP. Both will activate cytoplasmic DNA sensors such as TLR9 and cGAS to trigger innate immune responses like IFN response and inflammatory cell death (17–19). These responses will then promote autophagy, regulatory T-cell (Treg) differentiation, DNA repair, the epithelial-to-mesenchymal transition, mitochondrial dynamics, and so on (19, 20).

Nevertheless, prolonged activation of these innate immune responses caused by accumulated cytoplasmic DNA will contribute to pathogenic lung inflammation and tumorigenesis (15). Moreover, studies have recently shown that the cytoplasmic DNA sensing pathways, such as the cGAS-STING pathway and Absent In Melanoma 2 (AIM2) inflammasomes pathway, are primarily responsible for the pathology of COVID-19 (21–23).. Therefore, in lung inflammatory diseases and lung cancer, cytoplasmic DNA sensing can be a novel mechanism for investigating the inflammation-mediated immune defense (19). This review focuses on the dichotomous roles of cytoplasmic DNA-sensing pathways in pulmonary inflammatory disease and lung carcinoma, emphasizing the pro-inflammatory response, anti-tumor immunity, and tumorigenesis. We first briefly introduce the broad function of several types of cytoplasmic DNA sensing pathways to better understand their mechanism in lung diseases. Finally, we discuss the profound significance of these pathways for new therapeutic approaches.

## 2 Cytoplasmic DNA sources: A danger signal

**Figure 1:**
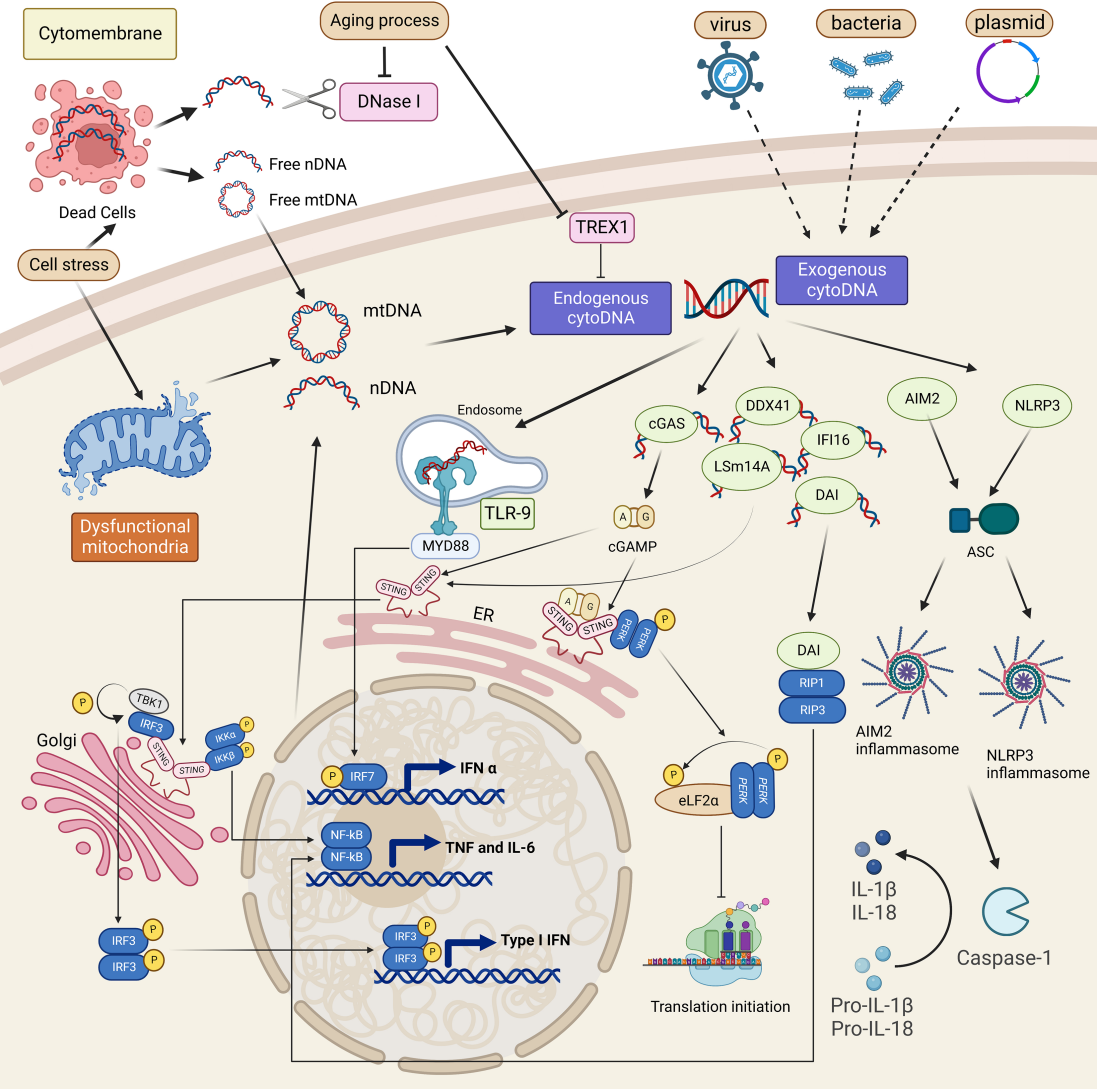
Cytoplasmic DNA sources and sensing pathways overview. Cell stress is the cause of mitochondria and nucleus dysfunction, which leads to self-mitochondria (mtDNA) or self-nuclear (nDNA) release into the cytoplasm. It also triggers cell death by releasing these two kinds of DNA into the extracellular matrix. Recognizing such cytoplasmic DNA, which is self or derived from dead cells and pathogens, cytoplasmic DNA sensors such as TLR-9, cGAS, DAI, DDX41, IFI16, and LSm14A will activate related immune responses, mainly the induction of type I IFN or TNF signaling. Inflammasomes are also involved in cytoDNA sensing and promote the maturation of IL-1β and IL-18. A non-canonical cGAS-STING pathway is also shown to promote the transcription of TNF-α through PERK.

DNA is typically stored in specialized compartments in eukaryotic cells. Both exogenous and endogenous DNA fragments presented in aberrant cellular compartments like cytoplasm act as a danger signal to stimulate the cellular innate immune response. Innate immunity can be triggered by exogenous DNA caused by viral or bacterial infections (24). Exogenous DNA of these pathogens, such as DNA viruses, retroviruses, and bacteria, is delivered to the host cytoplasm through a process like endocytosis (25). Their localization inside cells, seen as PAMPs, permits the recognition by cytoplasmic DNA sensors and triggers the innate immune response (18). The pathogenic infection also promotes mitochondrial DNA (mtDNA) release by triggering cell stress. Therefore, it is recognized as DAMPs to activate the immune system (**Figure 1**) (25).

Additionally, inflammation that develops in the absence of exogenous pathogens, called sterile inflammation, is a cell-autonomous response in which endogenous cytoplasmic DNA mislocalized from nuclei and mitochondria is a vital contributor (**Figure 1**) (15). Endogenous cytoplasmic DNA is mainly derived from cellular stress and cell death like apoptosis, which are always accompanied by mtDNA fragments leakage and abnormal chromosomes released from nuclei (15, 16). In addition to the disordered DNA production, the accumulation of DNA fragments in the cytoplasm can also be caused by the low activity of cytoplasmic deoxyribonucleases (DNases), DNA-degrading enzymes, including DNase I, DNase II, and 3’ repair exonuclease 1 (Trex1, or DNAase III) (26). Genetic depletion of DNases and the aging process, which increases the frequency of damage-induced DNA break, are both responsible for the increased cytoplasmic DNA, raising the risk of several autoimmune diseases (15, 27–29). Despite the different origins, once recognized, cytoplasmic DNA species share common or similar signaling pathways, predominantly the cGAS-STING pathway (**Figure 1**) (30). Further studies have discovered multiple cytoplasmic DNA sensors and other pathways specifically triggered by different DNA fragments (15). These signaling pathways function in cell type-dependent patterns and interplay to build the host defense and also, when aberrantly activated, lead to pathogenic inflammatory diseases.

## 3 Cytoplasmic DNA sensing pathways

### 3.1 Canonical cytoplasmic DNA sensing pathways

Current studies of cytoplasmic DNA sensing pathways mainly focus on the induction of interferon (IFN) production (**Figure 1**). IFNs are a group of proteins with anti-viral activities classified into three types, type I IFN, type II IFN, and type III IFN (31, 32). Type I IFN, such as IFN-α and IFN-β, mainly activates proteins of the JAK-STAT1 pathway that promote the transcription of interferon-stimulated genes (ISGs) to perform their immune functions (33).

#### 3.1.1 TLR9 pathway

Among PRRs, the best-studied one is the Toll-like receptor (TLR) family, whose pathways have been investigated sufficiently so far (18). Other TLR family members always work in the plasma membrane to recognize lipoproteins. At the same time, TLR3, TLR7, and TLR9 can detect nucleic acids inside the cell, where the sensing mechanism of TLR9 is relatively more explicit (16, 34). Mainly expressed on the plasmacytoid dendritic cells (pDCs) and B lymphocytes, TLR9 recognizes CpG-rich DNA derived from the genomes of microbes (16, 35). TLR9 in the endoplasmic reticulum (ER) membrane, mainly detecting the unmethylated CpG motif, moves to endosomal compartments such as lysosomes that contain CpG DNA. In these endosomes, viral particles and bacteria cell walls will be broken down to release nucleic acids, which can be modified in such compartments and be recognized by TLR-9 (34). Once combined with DNA, the TLR9 complex recruits MyD88 and promotes the function of IFN regulatory factor 7 (IRF7) to promote the production of type I IFN, predominantly IFN-α (16, 26). Therefore, TLRP-9 is not a kind of cytoplasmic DNA sensor since its sensing roles occur in endosomes and is more precise to be considered as an intracellular sensor.

Moreover, self-DNA, including mtDNA and DNA with the CpG motif like microbes produced by dead cells caused by pathogen infection, can also be recognized by TLR9, which depends on the nucleotide length and sequence (26, 35). On the one hand, the sensing will promote the production of Th1 cytokines such as IL-12 to trigger the differentiation of naïve T-cells. Besides, such sensing pathways have been proven to reduce inflammation throughout the body (35). Generally, the TLR9-MyD88 pathway is critical for activating CD8^+^ T-cells. In addition, the TLR9-MyD88-type I IFN pathway is important in promoting the adaptive immune response during the pathogen invasion and controlling autoimmunity (36, 37).

#### 3.1.2 STING-dependent sensing pathway

The stimulator of interferon genes (STING) is the signaling molecule encoded by TMEM173 and essential for transcribing type I IFN or cytokines in response to cytoplasmic DNA (18). The STING-dependent DNA sensing pathway is gradually noticed for its function in the immune response.

Cyclic GMP-AMP synthase (cGAS) is identified as an important cytoplasmic DNA sensor (30, 38, 39). cGAS recognizes cytoplasmic DNA derived from pathogen or self-DNA and is activated to produce the second messenger cyclic GMP–AMP (cGAMP), which then activates STING located in the endoplasmic reticulum (ER) (18, 35, 40). cGAMP-STING complex transfers to ER-Golgi intermediate compartment and Golgi, where STING recruits TANK-binding kinase 1 (TBK1) and IκB kinase (IKK) (16, 40). TBK1 then phosphorylates IFN regulatory factor 3 (IRF3) by forming a trimeric complex with STING and IRF3. IKK activates the nuclear factor-κB (NF-κB) inhibitor IκBα, respectively regulating the transcription of IFN-β and NF-κB to secret pro-inflammatory cytokines such as IL-6 (40–42). The cGAS-STING-TBK1-IRF3 pathway can protect against pathogen infection and mediate the inflammation on the condition of cell stress and tissue damage, whose activation is closely associated with autoinflammatory and degenerative diseases (20, 43).

Besides cGAS, interferon-regulatory factors (DAI/ZBP1), DDX41, IFI16, and LSm14A recognize cytoplasmic DNA as dsDNA sensors like cGAS but directly promote the function of STING through TBK1-IRF3 pathway (18, 26, 40). Among them, DAI is more common in fibroblasts, with the Zα domain identifying Z-DNA, the conformer of B-DNA, which is the typical form of dsDNA in cells (44–46). DAI can also recruit receptor-interacting protein 1 (RIP1) and receptor-interacting protein 3 (RIP3) through RIP homotypic interaction motifs (RHIMs) to activate the NF-kB pathway, eventually triggering necrosis (16, 45, 47).

#### 3.1.3 Inflammasomes dependent sensing pathway

AIM2, a human PYHIN protein, can directly recognize dsDNA in the cytoplasm, which has the carboxy-terminal ligand-binding HIN domains and amino-terminal protein-protein interaction signaling pyrin domain (26, 48). The HIN domain can directly bind cytoplasmic dsDNA to relieve its autoinhibition to interact with apoptosis-associated speck-like protein containing a CARD (ASC), recruiting pro-caspase-1 to this complex called AIM2 inflammasome (48–50). The oligomerization of pro-caspase-1 in AIM2 inflammasomes then triggers the activation of caspase-1 that will promote the maturation of its substrates pro-IL-1β and pro-IL-18 into IL-1β and IL-18, finally inducing pyroptosis (16, 48). Furthermore, studies have shown that when it comes to dsDNA microbe infections like HSV1 and *Francisella novicida*, AIM2 can combine with pyrin and ZBP1 to form a multiple-protein complex called AIM2 PANoptosome to induce a new type of specific cell death, PANoptosis (51). The AIM2 inflammasome can restrict the replication of bacteria and is involved in the pathogenesis of autoimmune diseases, such as systemic lupus erythematosus (SLE) and lung tumorigenesis (52, 53). Therefore, AIM2 mainly recruits specific molecules to assemble inflammasomes, where type I IFN is uninvolved in contrast to the STING-dependent signaling pathway, such as the cGAS-STING pathway. However, these two pathways are both involved in protecting against *Francisella novicida* infection. The recognition of *Francisella novicida* by cGAS-STING increases intracellular bacteria clearance, which contributes to the production of cytoplasmic dsDNA and subsequently activates AIM2 inflammasomes (16, 49).

Another kind of inflammasome, NLRP3, can also respond to cytoplasmic DNA, especially mtDNA (49). Many researchers have found that the production of mitochondrial reactive oxygen species (ROS) or mtDNA after using NLRP3 agonists can lead to the activation of NLRP3, but the mechanism remains elusive (16, 49). Firstly, NLRP3 needs the induction of the NF-κB pathway by TLRs such as TLR4 to be transcribed (48, 49). Then, activated by mtDNA, NLRP3 assembles inflammasomes with a new serine-threonine kinase (NEK7), ASC, and pro-caspase-1 to promote the maturation of IL-1β and IL-18 in a pattern similar to AIM2 inflammasome signaling (16, 48, 54). Meanwhile, STING can also activate NLRP3 inflammasomes through potassium efflux (16, 54).

### 3.2 Non-canonical cytoplasmic DNA sensing pathways

#### 3.2.1 Non-canonical cGAS-STING pathway

Though STING is recognized chiefly for recruiting TBK1 and IKK, studies have shed light on non-canonical STING-mediated pathways and their sentinel roles in triggering the inflammatory response (55, 56). Recently, an alternative cGAS-STING pathway that directly activates PKR-like endoplasmic reticulum kinase (PERK) was identified in addition to the classical cGAS-STING-TBK1-IRF3 pathway (20). In this novel pathway, activated STING at the ER binds with the located kinase PERK *via* their interaction of intracellular domains. Thus, DNA sensing by cGAS leads to the PERK-dependent phosphorylation of translation factor eIF2α, an essential regulator of cap-dependent initiation of mRNA translation (57). This STING–PERK–eIF2α pathway inhibits the overall cap-dependent mRNA translation while upregulating specific pathways like TNF-α signaling to shift the translation program to a preferred inflammatory pattern.

Intriguingly, the STING-PERK pathway is highly conserved, and its occurrence precedes and parallels the activation of the TBK1-IRF3 axis (20). Nucleic acid sensing has been established to regulate many defined cellular metabolic processes such as autophagy (58), mitochondrial dynamics (59), and senescence (15, 60, 61), and it has been found that hyper-sensing of nucleic acid leads to disruption of cellular homeostasis (15, 16, 56, 62, 63). The non-canonical cGAS–STING–PERK signaling regulates translation, functioning as a critical regulator of cellular senescence (20). Intervening in this pathway can attenuate lung fibrosis (20), which is reported to be correlated with cGAS-STING signaling but independent of IRF3-IFN and is a typical symptom of STING-associated vasculopathy with onset in infancy (SAVI) (64–66). Therefore, the cGAS-STING-PERK pathway, with its function in cellular metastasis and protein homeostasis, provides promising therapeutic value and gives an insight into the evolutionary conservative cellular system of damage-sensing.

#### 3.2.2 Ku70: A novel cytoplasmic DNA sensor in innate immunity

Ku70, which forms a heterodimer with Ku80 and assembles with DNA-PKcs as the subunit of the DNA-dependent protein kinase (DNA-PK) complex, is an endogenous nuclear protein that participates in the non-homologous end joining (NHEJ) pathway to repair DNA double-stranded breaks (67, 68). Novel studies have revealed that Ku70 is also involved in several cellular activities in the cytoplasm, including recognizing cytoplasmic DNA to trigger innate immune response (69–73). Ku70 binds to dsDNA fragments in a sequence-independent manner adapted to its nucleic functions facilitating its role as a PRR (68, 71). The Ku70-involved mechanisms of cytoplasmic DNA sensing show a relatively conservative but cell-type-dependent pattern. The induction of IFN and inflammatory cytokines mediated by Ku70, KU complex, or DNA-PK in a STING-dependent or independent way was confirmed in multiple cell types (69, 72–75). The STING-IRF pathway acts downstream of Ku70 to induce type I and III IFNs and cytokines during infection or transfection when it senses exogenous DNA, like those of VACV (vaccinia virus) or HSV (herpes simplex virus) (69, 74, 75). In addition, a recent study suggests that the accumulated cytoplasmic DNA in aged CD4^+^ T-cells sensed by the KU complex initiates the recruitment of DNA-PKcs, whose activation can be further promoted by TCR stimulation and phosphorylates the kinase ZAK. The ZAK-mediated AKT and mTOR pathways then promote T-cell activation and proliferation, which further contributes to the development of autoimmunity in aged mice (70).

## 4 Cytoplasmic DNA: Roles in the development of lung inflammatory diseases

Cytoplasmic DNA sensing is closely related to the lung immune response. Recent studies have shown that accumulated cytoplasmic DNA, including pathogen-derived DNA and self-DNA, gives rise to lung inflammatory diseases. Common acute pneumonia associated with the conditions mentioned above includes acute respiratory distress syndrome (ARDS), IFN-dependent acute lung inflammation, and respiratory infection from bacteria or viruses, in which neutrophils are often involved (76). With the accumulation of cytoplasmic DNA, acute lung inflammatory diseases develop gradually into chronic diseases, mainly divided into three categories, chronic obstructive pulmonary disease (COPD), progressive lung diseases including asthma and emphysema, and interstitial lung diseases (ILD). Here, the role of cytoplasmic DNA sensing in driving the function of immunity and the pathology of the lung diseases mentioned above will be illustrated by giving examples (**Table 1**).

**Table 1:** The mechanism of cytoplasmic DNA sensing in several kinds of lung inflammatory diseases.

Type of lung inflammation	The name of diseases	Proposed trigger	Pathways involved	Immune response	References
Acute lung inflammation	Acute respiratory distress syndrome (ARDS)	Pathogenic nDNA; self nDNA; mtDNA	cGAS-STING-TBK1; TLR9-MyD88-IRF7; NLRP3	IFN-1; IL-β/IL-18	(54, 77–80)
	Respiratory infection	Virus DNA; self nDNA	cGAS-STING-TBK1; TLR9-MyD88-IRF7;Th2;mo-DCs; AIM2 PANoptosome	IFN-1;CCL2/CCL7; PANopoptosis	(16, 51, 81–84)
Chronic lung inflammation	Asthma	Virus DNA; self nDNA	mo-DCs; cGAS-STING; Ku70	Type 2 immune response; IFN-1	(16, 85–87)
	Chronic obstructive pulmonary disease (COPD)	mtDNA; nDNA	TLR9-MyD88; cGAS/DDX41/IFI16-STING; NLRP3	IFN-1; IL-β	(16, 88–90)
	Idiopathic pulmonary fibrosis (IPF)	mtDNA; miRNA	STING; NLRP3; AIM2 inflammasome	IFN-1; IL-β	(16, 91–95)
	STING-associated vasculopathy with onset in infancy (SAVI)	TMEM173 mutant	/	Increasing the transcription of interferon-stimulated genes (ISG)	(66, 96–98)
	COPA syndrome	Missense mutations in the COPA gene	the traffic of STING back to ER from Golgi(cGAS-STING)	IFN signal	(43, 97, 99)

nDNA, nuclear DNA; mtDNA, mitochondria DNA; Th2, T helper 2 cell; mo-DCs, monocyte-derived dendritic cells.

### 4.1 Acute lung inflammation

#### 4.1.1 ARDS

IFN-dependent acute lung inflammation shows an indivisible relationship with STING. Silica, smoking, and pathogenic infection can induce cell death and release self-DNA, leading to the activation of the cGAS-STING pathway and driving IFN-dependent sterile inflammation (16, 77, 91). ARDS commonly happens in acute lung inflammation, resulting from the dsDNA caused by viral and bacterial infection (76, 78). Sterile inflammation can also contribute to the occurrence of ARDS. A low dose of non-nucleotidyl STING agonist diABZI in the bronchoalveolar cavity leads to the activation of the downstream TBK1-IRF3 pathway. In addition, it triggers the release of dsDNA from neutrophils through NETosis, the specific form of neutrophil cell death by the formation of neutrophil extracellular traps (NETs), or from the pulmonary cells through PANoptosis by the formation of PANoptosome ASC-Caspase 8-RIPK3 complex formation, which all causes ARDS (77).

Furthermore, self-DNA releasing stimulates cytoplasmic DNA sensors, mainly TLR9, DDX41, and IFI204, to initiate the secondary IFN I response in ARDS (77). Except for the abovementioned situations, the mtDNA in the cytoplasm is another induction factor of ARDS. Once oxidized mtDNA (Ox-mtDNA) produced after being exposed to reactive oxygen species on the loss of mitochondria membrane potential is cleaved by endonuclease FEN1 or repaired by DNA glycosylase OGG1 in mitochondria into about 600 bp fragments, it will be transported to the cytoplasm *via* mitochondrial permeability transition pores (mPTP) and voltage-dependent anion channel (VDAC) (54). Cytosolic Ox-mtDNA will then bind to and activate NLRP3 inflammasomes to promote the secretion of IL-1β and IL-18 that induce the production of IFN-γ and other cytokines or chemokines, leading to ARDS (79, 80).

#### 4.1.2 Respiratory infection

Pneumonia, presenting as an acute lower respiratory infection, is severe and commonly caused by viruses, bacteria, and parasites (76). The invasion of the pathogen breaks the homeostasis of the airway epithelium, the first barrier of the host, leading to the direct induction of immune cells from pathogens and triggering lung inflammation. Pathogen-derived nucleic acids from bacteria, such as *Mycobacterium tuberculosis*, viruses like herpes simplex virus 1 (HSV-1) and EBV (DNA viruses), stimulate cytoplasmic sensing pathways, including the cGAS-STING pathway and TLR9 pathway to trigger IFN I-mediated lung inflammation (16, 81). In addition, most RNA viruses, such as rhinovirus (RV), mainly induce host damaging and host dsDNA releasing through NETosis, which then activates STING-dependent cytoplasmic DNA sensing pathway (100). These responses induce T helper 2 cells to recruit monocyte-derived DCs (mo-DCs) by promoting the secretion of chemokines CCL2 and CCL7. This type 2 immune response occurs accordingly and always leads to allergic asthma exacerbation, a chronic lung inflammation (16, 82, 83).

Additionally, inflammasomes also play important roles in such epidemic infections. For example, in the infection of HSV-1 and *Francisella novicida*, AIM2 PANoptosome, whose assembly is driven by AIM2, pyrin, and ZBP1, can mediate PANoptosis, resulting in inflammatory cell death (51). However, AIM2 in influenza virus (IAV) infection was shown to mainly inhibit the excessive inflammatory response, though it is also promoted by dsDNA released by virus-infected necrotic bronchiolar epithelial cells and NETosis, showing the function of AIM2 as an inflammation regulator (16, 84).

### 4.2 Chronic lung inflammation

#### 4.2.1 Asthma

In chronic lung inflammatory diseases, eosinophils work dominantly (76). Asthma presents as chronic bronchial inflammation, mainly resulting from the mucous hypersecretion in bronchial walls due to IgE-mediated hypersensitivity (85). Eosinophils play vital roles in the previously mentioned potential pathogenesis for asthma, the excessive type 2 immune response (86). Besides pathogen infection, house dust mite (HDM) is another common allergen for asthma. HDM recruits eosinophils and neutrophils to produce reactive oxygen and nitrogen species (RONS) that triggers DNA damage, followed by the cGAS-STING-mediated IFN I response (16, 87).

#### 4.2.2 COPD

Besides asthma, COPD is one of the most common chronic lung diseases with the symptom of exertional dyspnea (85). Cigarette smoking is the main inducement of COPD, which promotes the DAMPs release, such as mtDNA and nuclear DNA from the necroptotic cell death. dsDNA is recognized by TLR9, cGAS, DDX41, and IFI16 that stimulate IFN I response, causing emphysema (16, 88, 89). In addition, NLRP3 inflammasome is activated by DAMPs to secret IL-1β and results in COPD (16). A new intermediate phenotype ACOS, asthma-COPD overlap syndrome, is recently reported that its mitochondrial dysfunction with a higher proportion of mtDNA/nDNA makes the phenotypes of ACOS close to COPD (85, 90).

#### 4.2.3 IPF

In idiopathic pulmonary fibrosis (IPF), a chronic, progressive interstitial lung disease, mtDNA is an essential prognostic marker whose mechanism remains further explored (4). Like COPD, environmental factors such as cigarettes and silica can lead to IPF. For instance, silica in the airway will induce ROS release, which contributes to the death of epithelial cells and immunocytes, including macrophages and neutrophils, with the release of dsDNA such as mtDNA, which then activates STING (16, 92). In addition to activating IFN I response, mtDNA and STING can also play a role in activating NLRP3 inflammasome, promoting IL-1β secretion and leading to emphysema and ILD (16, 91).

#### 4.2.4 SAVI and COPA

Monogenic autoimmunity diseases are also common in abnormal cytoplasmic DNA sensing processes. STING-associated vasculopathy with onset in infancy (SAVI) is a new type of type I interferonopathies commonly seen in infants (16, 96). SAVI patients develop fibrosis rapidly in the early stage and show symptoms of ILD simultaneously. Current studies have found that SAVI is usually caused by the mutation of the STING coding gene TMEM173, in which TMEM173 N153S mutant mice are the most researched (66, 96). It was found that TMEM173 N153S mutation triggers lung inflammation independently of cGAS and IRF3, which mainly increases the phosphorylation of STAT1 in T lymphocytes, thereby increasing the transcription of ISG such as *Cxcl10* and leading to autoimmune disease (96–98, 101, 102). In addition, like SAVI, COPA syndrome derives from missense mutations in the COPA gene encoding the COPα protein subunit of the COPI complex, which regulates the transportation of STING in the ER (97, 99). In COPA, most patients also show ILD; the onset period is mainly in childhood, slightly later than SAVI (97). COPA mutation reduces STING traffic back to ER from Golgi after identifying cGAMP, which results in continuous STING signaling and increased IFN induction (43, 99).

### 4.3 COVID-19

The manifestations of COVID-19 are mainly pulmonary pathologies and extrapulmonary complications, with symptoms ranging from asymptomatic to severe clinical outcomes and death (103). Severe COVID-19, accompanied by a wide range of complications, always leads to fatal diseases, including pneumonia, ARDS, or long-lasting visceral injury beyond the lung (104). Many characteristics of severe COVID-19 have been reported with clinical outcomes (105–110). One significant hallmark of severe or critical COVID-19 is the defective type I IFN responses and elevated production of pro-inflammatory cytokines or chemokines (23, 111). The rapid rise of IFN I and IFN-λ (type III IFN) in the early infection stage is critical for the balance of immune response since it induces the anti-virus state in cells (107). This process typically precedes the pro-inflammatory response (112). A temporal analysis of IFN and major inflammatory cytokine patterns in patients with moderate-to-severe COVID-19 reveals that both type I and type III IFN are diminished and delayed during the early infection. In contrast, the production of pro-inflammatory cytokines such as IL-6 and TNF occurs before IFN, and the high concentrations are maintained for a prolonged time (22). This untuned anti-virus response leads to a hyper-inflammatory state known as the cytokine storm. The evasion of SARS-CoV-2 from the host anti-viral immune response well accounts for the diminished IFN production (113, 114). In addition, mutations and autoantibodies that interfere with IFN-related pathways, including virus RNA sensing, IFN production, and response, have been identified in severe COVID-19 cases and shown to be correlated with poor outcomes (115, 116).

The dual role of type I IFN in the anti-viral response and immunopathology has been well described. Higher IFN-λ was reported to correlate with lower viral load and faster clearance in the respiratory tract and reduce the severity of COVID-19 in the upper airways (22, 117). In contrast, despite its protective role in the early infection phase to limit virus proliferation, a sustained high level of IFN I in the late phase enhances the aberrant inflammation and is associated with high complication risk and poor clinical outcomes (23, 106, 117–119). Notably, induced IFN production was observed only in a fraction of patients who became critically ill with augmented inflammation (22). They exhibited stronger innate immune and anti-viral responses characterized by increased pro-inflammatory mediators compared to healthy and non-critically ill patients. Inflammasome genes and PRRs responsible for microbial recognition, such as AIM2, were also upregulated, contrary to the enhanced anti-inflammatory response in non-critically ill patients (22). The IFN-mediated immune dysregulation in severe COVID-19, accompanied by lymphopenia and high neutrophil counts, was reported to result in worse disease cases (103, 106). Clinical administrations warrant a tight regulation of IFN I in case of its collateral damage. However, the underlying mechanism that regulates or maintains IFN I expression remains elusive.

Recent studies show that the cGAS-STING pathway plays a vital role in the induction of IFN-dependent aberrant inflammation in COVID-19 (23, 110, 120). Like all plus-strand RNA viruses whose replication processes do not produce DNA species, SARS-CoV-2 can be recognized directly by RNA sensors such as RIG-I-like receptors (RLRs) and TLRs, or indirectly through potent DAMPs such as the provoked accumulation of cytoplasmic DNA (121). Here we focus on the later. Domizio et al. demonstrate that cGAS-STING-activated IFN I response is prominent in the damaged lung tissues of severe COVID-19 patients. Beyond the lung, STING-depended IFN I signal is also detected in skin lesions of patients with mild-to-severe COVID-19 along with high levels of ISGs and pro-inflammatory cytokines. Endothelial cells and perivascular macrophages in both tissues are established to mediate the aberrant cGAS-STING response, each with a distinct cellular process. SARS-CoV-2 can induce mitochondria dysfunction, and the endogenous mtDNA released activates cGAS-STING within endothelial cells, inducing IFN I production, cell activation, and death (23, 122–124). Regarding macrophages adjacent to the damaged areas, the cGAS response derives from recognizing DNA from engulfed dying endothelial cells (23). In addition, the nuclear rupture caused by the fusion of SARS-CoV-2-infected pneumocytes is also reported to be accompanied by DNA leakage into the cytoplasm, which subsequently activates cGAS and STING (120).

The high level of pro-inflammatory cytokines in severe COVID-19 cases is interpreted by another study which demonstrates cGAS-STING signaling triggered by SARS-CoV-2 infection contributes to the NF-κB-mediated production of cytokines in human epithelial cells, probably driving aberrant inflammatory response in patients (110). Notably, the activation of cGAS-STING is not canonical in this case since a restriction of IRF3 nuclear transcription and subsequent IFN I induction was reported from the transcriptomic profiles, and cytokines profiles of SARS-CoV-2 infected lung epithelial cells showed a lack of type I and III IFNs, consistent with the findings of Domizio et al. (23)

An overview of the inflammatory response during severe COVID-19 could be formed. In the early infection stage, respiratory epithelial cells are first attacked. Due to the evasion mechanism of SARS-CoV-2, IFN induction is blocked within infected cells. At the same time, an increase of NF-κB-mediated cytokine production is induced, leading to a lung profile with a preference for inflammatory response. During the pathogenesis of severe COVID-19, disrupted mitochondrial homeostasis in vascular endothelial cells adjacent to the infection sites causes the accumulation of mtDNA in the cytoplasm, which initiates the cGAS-STING-IFN I response and the ultimate cell death. The ensuing engulfment of dying endothelial cells and the recognition of cytoplasmic DNA trigger the production of IFN I and cytokines within perivascular macrophages. This, along with the NF-κB-mediated pro-inflammatory response and chemokines likely induced by prolonged IFN I secretion that recruits immune infiltrates (125), further contributes to the cytokine storm in severe COVID-19 cases. The enhanced inflammatory response gives rise to more severe respiratory diseases in patients.

Some ISGs upregulated by IFN signaling may also be involved in deteriorating the case. For instance, ZBP1 was identified as a critical ISG and a cytosolic sensor to drive PANoptosis when administered with IFN therapy during β-coronavirus infections (126). Sensing Z-nucleic acid through the ZBP1 Zα domain enables ZBP1 to interact with RIPK3 and recruit caspase-6 and caspase-8, triggering an NLRP3-mediated anti-viral response and pyroptotic cell death (51, 127). NLRP3 inflammasome was also reported to sense coronavirus infection (128, 129). Besides, colonization of NLRP3 and AIM2 with ASC specks was observed when co-stained in monocytes and lung macrophages but not in lung epithelial cells of patients with COVID-19 (21). It indicates that inflammasome-mediated cell death aborts the infection of invading virus, but inflammatory cytokines released would cause systemic inflammation. However, the specific mechanism of AIM2 as a cytoplasmic DNA sensor remains elusive in this case. Another study further supported the role of AIM2 by suggesting that AIM2 activation by cytosolic dsDNA is responsible for the IL-1α, IFN-α, and TGF-β release from circulating monocytes of patients with prolonged COVID-19 symptoms, likely contributing to the risk of developing lung fibrosis as a sequelae (130).

## 5 Cytoplasmic DNA: Roles in the development of cancer

Lung cancer has a close relationship with lung inflammation. Chronic inflammatory signaling may lead to cancer development by simulating cellular proliferation and survival (131). Additionally, It has been observed that canceration usually occurs at the site of chronic inflammation, and there are numerous inflammatory cells found in the tumor (132). Chronic airway inflammation leads to the bronchial epithelium and lung microenvironment changes, resulting in the conducive development of lung cancer (132, 133). For example, studies have proved that COPD causes lung cells to be exposed to proinflammatory cytokines such as the NF-kB pathway, thereby increasing cell proliferation and leading to lung cancer (133, 134). To clarify the roles of cytoplasmic DNA in lung cancer, we primarily discuss the universal mechanisms that how cytoplasmic DNA is involved in anti- and pro-tumorigenic effects followed by its potential role in lung carcinoma development and propagation.

### 5.1 Anti-tumorigenic effects

**Figure 2:**
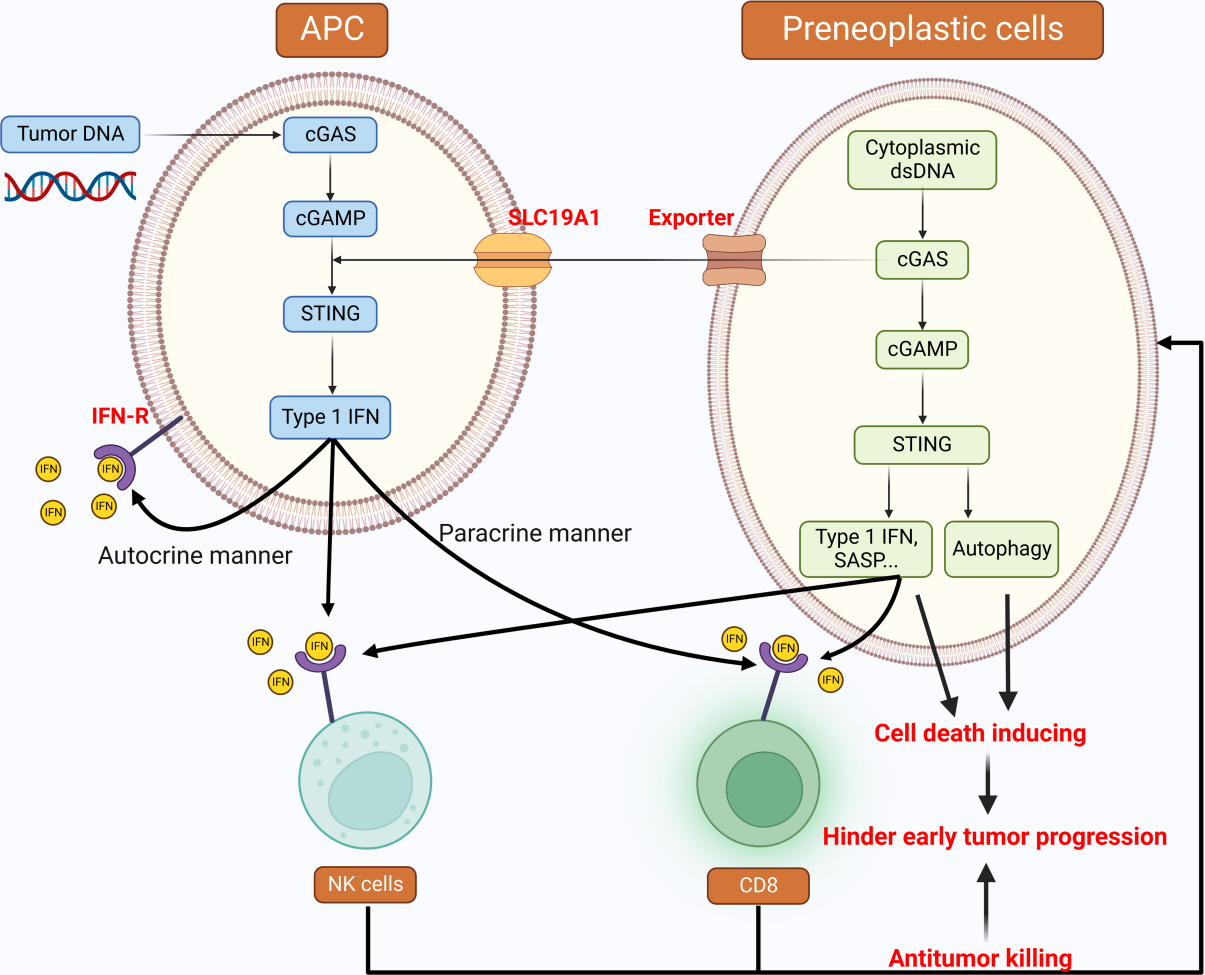
Cytoplasmic DNA: tumor suppressive roles in the development of cancer Cytoplasmic DNA sensing pathways implement anti-tumor responses in both tumor cell-autonomous and non-cell-autonomous manners. These pathways are activated by both cytoplasmic dsDNA inside the preneoplastic cells and tumor DNA engulfed by APCs. The downstream of type I IFN is the cross-priming of immune cells, such as NK cells and CD8+ T-cells to hinder early tumor progression. STING-mediated autophagy can also perform the tumor suppressing function. APC: Antigen-presenting cells, NK cells: Natural killer cells, CD8: CD8^+^ T-cells.

Different from normal cells, tumor cells are often filled with cytoplasmic dsDNA derived from viruses, mitochondria, genomes, and so on (19). Thus, innate cytoplasmic DNA sensing pathways, which can detect abnormal cytoplasmic DNA, play a vital role in anti-tumor responses in both tumor cell-autonomous and non-cell-autonomous manners. For the former, activation of the cGAS-STING pathway in tumor cells can upregulate a series of inflammatory genes, such as type I IFNs, to induce cell death. STING-mediated autophagy can also function as a hindrance to early tumor progression through an unknown mechanism (135). In addition, IFN signaling can promote tumor surveillance by recruiting and infiltrating immune cells like natural killer (NK) cells and T-cells (19, 61). Most importantly, the activation of this pathway also mediates the secretion of senescence-associated secretory phenotypes like pro-inflammatory cytokines and chemokines. The resulting immunostimulatory factors then restrict tumorigenesis through recruiting immune cells and clearing tumors (19, 61, 136). Apart from the cell-autonomous manner, the host may also harness inflammatory pathways for tumor surveillance in a non-cell-autonomous manner. Accumulated tumor DNA engulfed by antigen-presenting cells (APC) such as macrophages and DCs activate the STING-IRF3 pathway and IFN signaling (19, 137, 138). Previous studies have shown that type I IFN can, in turn, promote the activation and functional maturation of DCs, thereby facilitating potent antigen presentation to CD4^+^ T-cells and cross-presentation to CD8^+^ T-cells in a paracrine or autocrine manner for immunity (63, 139–141). Additionally, cGAMP induced by the cGAS pathway in preneoplastic cells can be secreted into the extracellular environment by a cGAMP exporter and imported into immune cells through the folate transporter SLC19A1 (142). This cGAMP subsequently activates the STING-IRF3 pathway and induces NK cell- and CD8^+^ T-cell-mediated anti-tumorigenic effects (**Figure 2**) (19, 143).

The characteristics of tumors with deficient mismatch repair (dMMR) ability include high tumor mutation burden, high sensitivity to immune checkpoint blockade (ICB) therapies, and potent responses in DC-mediated T-cell cross-activation (144). MLH1 is a vital protein of the MMR process and inhibits tumorigenesis by correcting biosynthetic errors during DNA replication (145). Recent studies showed that tumor models with MLH1 deficient (dMLH1) accumulate cytoplasmic DNA and produce IFN-β in a cGAS-STING-dependent manner due to failure to repair damaged DNA. This increases the infiltration of T-cells in dMLH1 tumors and responses to ICB therapies to limit tumor progression. In the clinic, downregulation of cGAS or STING in dMMR tumors is associated with poor prognosis since it decreases DNA-sensing-mediated anti-tumor immune surveillance. Thus, the level of cGAS/STING expression can predict survival in dMMR tumor patients (144). In a word, the STING-dependent signaling pathway can be activated in various cells such as T-cells, B cells, macrophages, NK cells, and some other leukocytes, therefore playing a central role in innate and adaptive immune responses that should be applied in cancer immunotherapy (146, 147).

### 5.2 Pro-tumorigenic effects

**Figure 3:**
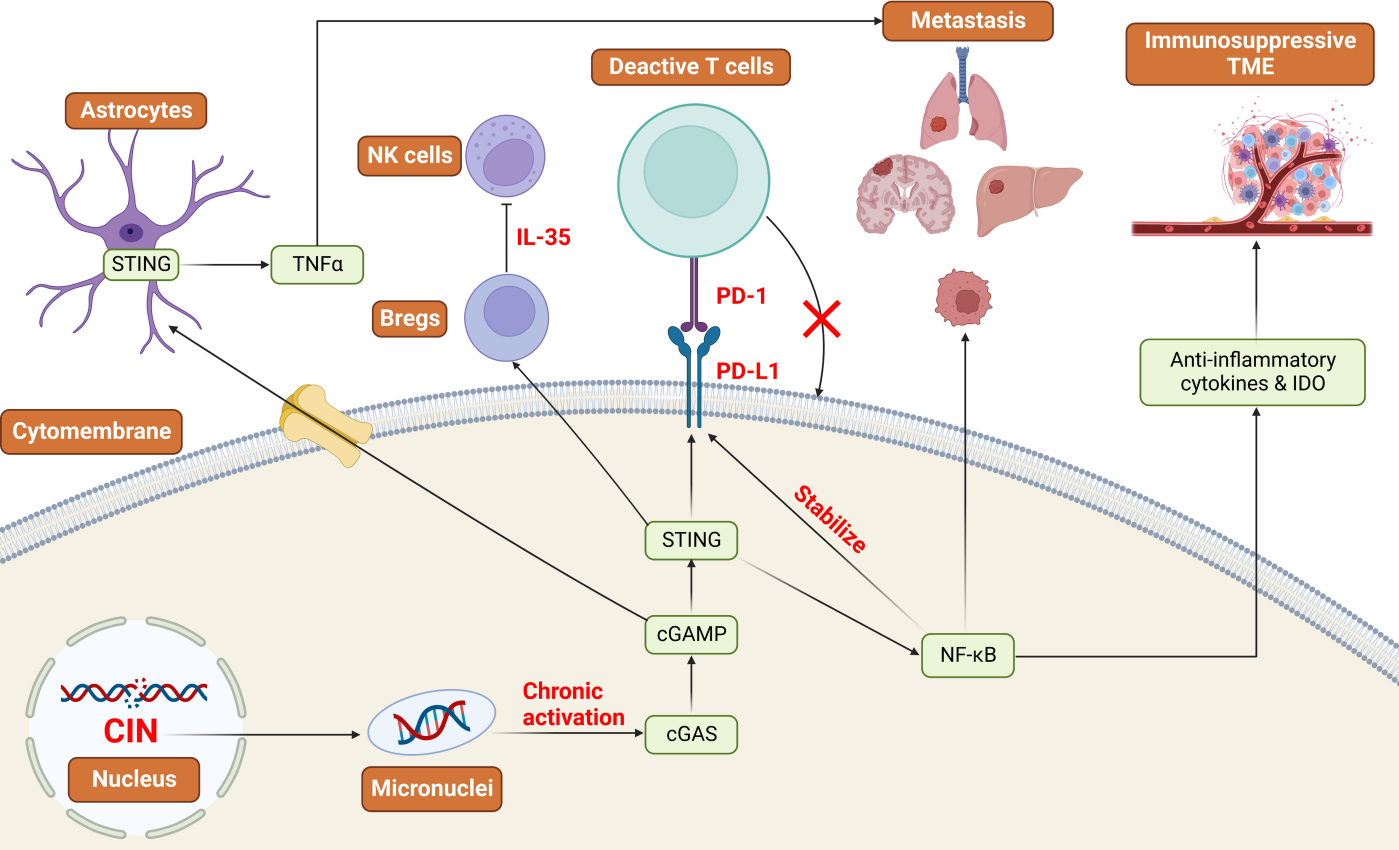
Cytoplasmic DNA: oncogenic roles in the development of cancer Tumors with high CIN produce micronuclei into the cytoplasm and chronically activate the cGAS-STING pathway. This will lead to the upregulation of NF-κB signaling pathways and the downregulation of IFN signaling pathways. As a result, NF-κB stabilizes the expression of PD-L1, and STING promotes the expression of PD-L1 to realize the immune escape. Additionally, NF-κB can promote tumor metastasis and the production of immunosuppressive TME. STING signaling pathways can also promote the expression of PD-L1 and the functions of Bregs to implement immune suppression and evasion. CIN: Chromosomal Instability, IDO: Indoleamine 2,3-dioxygenase, PD-L1: Programmed Cell Death Ligand 1, PD-1: Programmed Cell Death Protein 1, TME: Tumor microenvironment, NK cells: Natural killer cells, Breg: Regulatory B cells.

Since cGAS-STING-mediated IFN signaling can limit tumor growth, tumor cells may silence this pathway to evade immune surveillance. Intriguingly, decreased methylation of cGAS and STING promoters was observed in most tumors, indicating that tumor cells do not normally grow by silencing the cGAS-STING pathway (148). Abundant evidence suggests that cytoplasmic DNA sensing pathways have dichotomous roles in tumorigenesis. They can also have pro-tumorigenic effects, such as promoting tumor metastasis and immune escape depending on the specific context and the stage of tumor progression (**Figure 3**). As described above, they lead to immune surveillance and tumor cell senescence during the early steps of tumor progression. Paradoxically, they will induce an immune-suppressive microenvironment if chronically activated (19).

Recent work has demonstrated an important link between chromosomal instability (CIN) and tumor metastasis. CIN resulting from replication errors in chromosome segregation during the mitosis phase is suggested to be a primary source of cytoplasmic DNA, a hallmark of cancer, and is associated with tumor progression (19, 62). Tumors with high CIN produce micronuclei which rupture and release DNA into the cytoplasm. Given that these tumors are flooded with cytoplasmic DNA, this leads to the chronic activation of the cGAS-STING pathway and upregulation of downstream inflammatory responses like NF-κB signaling that favor tumor invasion and metastasis (62), together with inhibition of IFN, which performs anti-tumorigenic effects normally (149). In addition, immunostimulatory molecules such as anti-inflammatory cytokines and indoleamine 2,3-dioxygenase (IDO) are released from the tumor with the activation of non-canonical NF-κB signaling, allowing the formation of an immunosuppressive microenvironment (19).

Furthermore, NF-κB signaling has been verified to stabilize the expression of Programmed Cell Death Ligand 1 (PD-L1), which is a kind of transmembrane protein combined with Programmed Cell Death Protein 1 (PD-1) to promote metastasis and evade immune surveillance by restraining the apoptosis of Tregs and activating apoptosis of antigen-specific T-cells (19, 150, 151). Activated STING also promotes the induction of PD-L1 and immunosuppressive cytokines such as IL-10 and CCL22, suppressing the immune responses by facilitating the infiltration of Tregs (152, 153). Besides the cell-autonomous manner, tumor cells can accelerate tumor progression by transferring cGAMP to neighboring astrocytes *via* gap junctions. Then the ensuing activation of STING promotes the secretion of TNF-α, a member of the TNF/TNFR cytokine superfamily from astrocytes involved in tumor metastasis (19, 154). In addition, the latest research has revealed that STING signaling activates regulatory B cells (Breg) function by inducing IL-35 to inhibit the anti-tumor effects of NK cells (155).

Some studies revealed other pro-tumorigenic effects of cytoplasmic DNA sensing pathways in specific cancer types. For cutaneous carcinoma, DNA damage in keratinocytes triggers STING-mediated production of cytokines such as IL-1, which can bind to its receptors TLRs in paracrine to phagocytes or autocrine. After binding, additional cytokines are produced *via* the MYD88 adaptor molecular and further drive the propagation of inflammation and tumor growth (63). Recent studies on triple-negative breast cancer (TNBc) suggest that TNBc cells normally encounter apoptosis mediated by the STING-IFN-STAT1 signaling pathway. However, CIN facilitates tumor cell survival by triggering IL-6-STAT3-mediated signaling through the cGAS-STING and NF-κB pathways (149). IL-6 produced following the activation of NF-κB signaling also suppresses the STING-IFN-STAT1 signaling (149, 156, 157). Clinical blockade of IL-6 signaling by the Tocilizumab, which targets the IL-6 receptor, can inhibit the IL-6-STAT3 signaling and hinder tumor progression (149). As for the cancers related to intestinal cells, one possible reason that causes tumorigenesis is the chronic wound without repair. IL-1β and IL-18 are secreted following the activation of the STING signaling by damaged intestinal cells to facilitate wound repair. However, suppose the wound does not experience repair; in that case, concomitant inflammations will alter the microbial composition in the gut to ones with genotoxic capacity and further lead to DNA damage and tumorigenesis (63, 141, 158).

In summary, cytoplasmic DNA sensing pathways perform pro-tumorigenic functions if chronically activated, such as in tumors with high CIN. This effect is mostly caused by the upregulation of NF-κB signaling and the downregulation of IFN signaling (**Figure 3**). A key unknown question is how tumors alter the downstream signaling of STING from IFN to NF-κB to adopt metastatic behavior (19). One hypothesis is that this regulation is related to multiple factors, such as the genomic states, tumor progression, and whether the downstream effector programs work correctly.

### 5.3 Roles in lung cancer

As mentioned before, lung cancer is the leading cause of cancer deaths in the world, of which approximately 85% of patients are known as non-small cell lung cancer (NSCLC), mostly including lung adenocarcinoma (LUAD) and lung squamous cell carcinoma (LSCC) subtypes (9, 159). Among various genetic mutations that cause NSCLC, some primarily mutated genes can influence cytoplasmic DNA sensing pathways and promote tumorigenesis. We will then illustrate the mechanisms based on different mutation types.

#### 5.3.1 TP53 mutant type

The most common mutation found in LUAD is TP53 (~46%) (160), a suppressor gene encoding the P53 protein. Mutated P53 functioning as a pro-tumorigenic effector promotes tumor cell survival and evasion surveillance of host cells in both cell-autonomous and non-cell-autonomous manners. To be specific, mutated P53 binds to TBK1. It prevents the trimeric complex formation between STING, TBK1, and IRF3, which is required to activate IRF3 and downstream signaling to initialize the innate immune responses. Therefore, mutated P53 alters cytokines production and leads to immune evasion by inactivating innate immune signaling (41).

#### 5.3.2 LKB1 mutant type

Mutated LKB1 (STK11) was observed in about 17% of patients with LUAD (160). LKB1 is the main upstream activator of AMPK, a suppressor of mTORC1, and targets defective mitochondria for autophagy (161). Thus, lung cancers with LKB1 absence develop mitochondrial dysfunction and a growth advantage due to unrestricted mTOR signaling (162). The pathologic accumulation of cytoplasmic mtDNA is related to mitochondrial dysfunction in LKB1 mutated tumors. LKB1 loss also results in apparent silence of STING expression and insensitivity to cytoplasmic DNA sensing, which is at least mediated by the hyperactivation of DNMT1 and EZH2 activity and reinforced by the upregulation of DNMT1 (163). In this case, the phenotype of marked inhibition of STING in LKB1 mutated tumors is coupled with selection pressure to avoid the deleterious impact of cytoplasmic mtDNA release (163).

#### 5.3.3 KRAS mutant type

Patients with mutations of KRAS, the most frequent isoform of mutated proto-oncogene RAS, account for about 33% of the total LUAD population, second only to TP53 and higher than LKB1 (160, 164). KRAS-driven NSCLC frequently inactivates TP53 or/and LKB1, of which KRAS-LKB1-mutant (KL) ones are particularly aggressive. KL tumors lack the expression of PD-L1 and downregulate chemokines that promote T-cell recruitment. Thus, they respond poorly to ICB therapies such as PD-1/PD-L1 (165–168). As described above, STING promotes the downstream TBK1-IRF3 signaling activation, producing IFN and STAT1-related programs to protect cells from abnormal states (63, 163). Without STING in KL tumors, TBK1 cannot combine with IRF3 (41). Therefore, this favors NF-κB-related signaling and the secretion of certain cytokines like IL-6, then the activation of IL-6-STAT3 signaling to promote bone marrow cell recruitment and tumor cell survival (149, 165, 169). Since the cGAS and cGAMP are maintained in the KL tumors with the restriction of STING expression, the accumulation of cGAMP can promote tumor metastasis by transferring to neighbor cells, which further contributes to KL tumor pheotypes (154, 170).

#### 5.3.4 SOX2 overexpression type

In addition, SOX2 overexpression is crucial in promoting undruggable LSCC (171, 172). SOX2, a crucial transcription factor, is the cytoplasmic DNA sensor in neutrophils, particularly in identifying bacterial genomes (173). After binding with dsDNA, SOX2 is suggested to activate the kinase TAK1 and its binding partner TAB2 (Tab2/TAK1 complex) to trigger the NF-κB pathway, thus may lead to tumor growth and metastasis (18, 173).

## 6 Cytoplasmic DNA: Applications in treating lung inflammatory diseases and cancer

### 6.1 Application in lung inflammatory diseases

#### 6.1.1 Promote anti-viral responses

Most kinds of lung inflammatory diseases such as respiratory infections, IPF, and COVID-19 are severe and nearly fatal, whose pathology of cytoplasmic DNA sensing is complex (1, 5, 174). For infectious lung diseases, the requirement for inflammation response varies during different stages of infections (15). At the beginning of a pathogenic infection, promoting anti-viral responses is more important than attenuating inflammatory responses (**Table 2**). Studies have shown that applying lipopolysaccharide (LPS) or ATP in cells with dysfunctional mitochondria increases the amount of mtDNA in the cytoplasm and then activates NLRP3 inflammasome to promote the secretion of IL-β or IL-18 (15). For stimulating the STING-dependent sensing pathway, STING agonists such as PC7A, alum and chitosan can promote the formation of STING–PC7A condensates that induce more sustainable production of cytokines than cGAMP-STING (16, 122, 175). Nevertheless, more STING agonist is used for oncology which is discussed later (16).

**Table 2:** Potential therapy for lung inflammatory diseases (including COVID-19) through regulating cytoplasmic DNA sensing pathway.

	Target	Therapies	Consequence	Immune response	Indications	References
Promote anti-viral responses	Cytoplasmic DNA source	LPS/ATP	Cytoplasmic mtDNA increasing	NLRP3 inflammasome		(15)
	Sensing pathway	STING agonist such as PC7A/alum/chitosan	Formation of STING-PC7A	Produce of cytokines increasing	Mainly for cancer; COVID-19	(122, 129, 175–177)
Attenuate the inflammatory responses	Cytoplasmic DNA source	DNase I	Accumulation of cyto-DNA decreasing	Type 2 immune responses alleviating	Asthma	(16, 82)
		FEN1-IN-4/VBIT-4	Release of mtDNA inhibition	IFN response decreasing	ARDS	(15, 54)
	Sensing pathway	PF-06928125/G150/hydroxychloroquine	cGAS inhibiting	cGAS-STING pathway inhibiting	SAVI; COPD; respiratory infections	(43, 178–180)
		Astin C/nitro fatty acid/C-178 and Cys91	STING inhibiting	STING dependent pathway inhibiting	SAVI; COPD; respiratory infections	(43, 122, 181–183)
		OLT1177/Colchicine	NLRP3/AIM2 inhibiting	Cell death decreasing	COPD; IPF; respiratory infections	(51, 80)
		ISGs interfering	ZBP1 deletion	PANoptosis blocking	COVID-19	(126)
	Downstream IFN pathway	Ruxolitinib/baricitinib/tofacitinib	JAK1/2 or JAK1/3 inhibiting	/	SAVI; COPA	(97)
		Anti-IL-6 therapies	/	/	COVID-19 (conflict with the clinical trail)	(129, 184–186)

LPS: lipopolysaccharide; In the indications part, the blue word means that the therapies have not been tested in such disease models and are only theoretical opinions.

#### 6.1.2 Attenuate the inflammatory responses

The inflammatory diseases introduced above are mostly autoimmune diseases that warrant attenuating the inflammatory responses. The treatment is mainly divided into three parts: inhibition of cytoplasmic DNA overproduction, cytoplasmic DNA sensing pathways, and the final immune function (**Table 2**).

It is mentioned above that the mutation of DNase like Trex1 or special DNA-releasing triggers like NETosis will lead to the accumulation of excess cytoplasmic DNA, which finally causes lung inflammation (15, 16, 82). Studies have shown that the administration of DNase I significantly alleviates type-2 immune responses in asthma from RV infection and STING-dependent lung inflammation caused by self-DNA accumulation in mice airways by limiting the accumulation of cytoplasmic DNA, suggesting its role in the treatment of such autoimmune lung diseases (16, 82). In addition, cytoplasmic mtDNA overproduction, which derives from the cleavage of ox-mtDNA by endonucleases like FEN1 and transportation of fragments to the cytoplasm, activates NLPR3 and gives rise to ARDS (54). FEN1 inhibitors like FEN1-IN-4 have been found to disrupt the release of mtDNA fragments. However, they do not affect the production of ox-mtDNA in mitochondria (54). Also, using VBIT-4 that inhibits the VDAC oligomerization has been shown to decrease mtDNA releasing and the level of subsequent IFN responses in a mouse model of systemic lupus erythematosus (15). It suggests that these two kinds of inhibitors can be new effective therapies to relieve lung diseases such as ARDS by reducing the presence of cytoplasmic mtDNA.

Another therapeutic idea is to intervene in the sensing pathway of cytoplasmic DNA. Many cytoplasmic sensors, such as cGAS, TLR9, and DDX41, have similar sensing patterns for DNA. Here we take cGAS-STING as an example. Several cGAS inhibitors (*e.g.*, PF-06928125 and G150) inhibiting catalytic sites and antimalarial drugs (*e.g.*, hydroxychloroquine) disrupting DNA binding have been developed (43, 178–180). STING inhibitors which mainly target STING CDN-binding sites (*e.g.*, Astin C) or palmitoylation sites (*e.g.*, nitro fatty acid) were also investigated (43, 181, 182). Moreover, covalent small molecule inhibitors, including C-178 and Cys91, are special STING inhibitors that can form covalent binding with each other. Such binding can especially disrupt STING-dependent pathways and show effective therapy results in the autoimmune mice model (122, 183). These cGAS or STING inhibitors potentially treat lung inflammatory diseases such as SAVI, COPD, and respiratory infections, while further clinical tests are required (16, 122).

Inflammasomes also play essential roles in cytoplasmic DNA sensing. NLRP3 inflammasome is crucial for the pathogenesis of IPF, ARDS, and COPD (80). OLT1177, a selective inhibitor of NLRP3 inflammasomes, can significantly reduce the maturation and secretion of IL-β and IL-18. Its effect has been tested in cryopyrin-associated periodic syndrome (CAPS). Therefore, its application is prompted in the treatment of COPD or IPF (80). AIM2 is another important inflammasome that can trigger a new kind of cell death called PANoptosis with ZBP1 and pyrin, resulting in respiratory infections from HSV1 and *F. novicida*. Colchicine, a drug inhibiting pyrin activation, can decrease the levels of cell death and the secretion of IL-β and IL-18. Thus, it may be a new potential drug for treating such programmed inflammatory cell death from the infections of these two pathogens (51).

Ultimately, inhibition of the downstream IFN pathway reduces inflammation responses, though the efficacy is relatively lower (122). JAK1/2 inhibitors like ruxolitinib or baricitinib and JAK1/3 inhibitors like tofacitinib have been proven to improve the pulmonary phenotype in SAVI and COPA patients, but only in milder conditions (97). It also suggests the potential importance of early diagnosis and intervention (97). Besides these two diseases, such targeted therapy can also apply to other interstitial lung diseases, which require further clinical trial (43, 56, 122).

#### 6.1.3 Therapies for COVID-19

Due to the unbalanced IFN levels in the serum of severe COVID-19 patients, the effect of IFN-based therapies is limited mainly to the early stage of infection and only shows prophylactic potential (176, 187, 188). Therefore, determining the optimal time frame to administer IFN is vital for therapeutic efficacy. Additionally, stimulation of endogenous IFN by agonists engaging in nucleic acid sensing pathways shows clinical benefits beyond exogenous administration (**Table 2**). For instance, treatment with the STING agonist was found to block SARS-CoV-2 infection (129, 176, 177). Besides optimizing anti-viral responses, immunomodulatory strategies that aim to attenuate inflammatory responses have been considered due to the pathogenicity of excessive cytokine production in severe COVID-19 (**Table 2**) (115, 126, 129, 189). Many anti-cytokine therapies, like anti-IL-6, have been evaluated, and the clinical trial results were conflicting, warranting more specific identification of pathologic stages and patient conditions (129, 184–186). Given the contribution of inflammatory cell death to the pathogenicity of COVID-19, the strategy of targeting pro-inflammatory signaling pathways is highlighted. As an example, interfering with ISGs may be considered a potential combined therapeutic choice since deletion of ZBP1, which blocks PANoptosis, was shown to inhibit the cytokine storm, lung damage, and lethality in infected mice during IFN treatment (126).

### 6.2 Application in cancer

**Figure 4:**
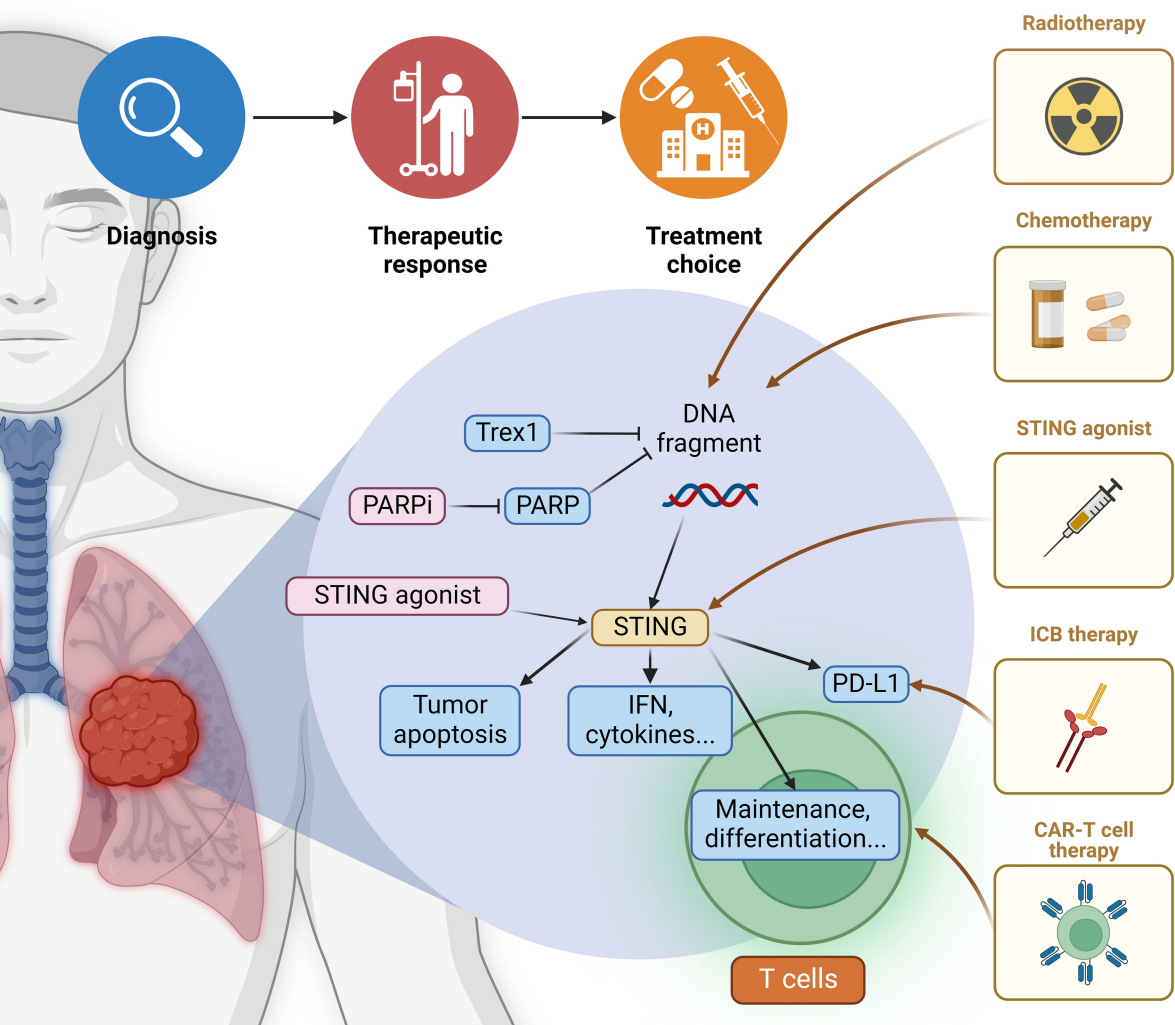
Application of cytoplasmic DNA in lung cancer therapies Current cytoplasmic DNA-related cancer therapies mainly focus on the promotion of cGAS-STING signaling. The specific therapeutic option should be determined based on the tumor conditions and basal level of STING activation. Common radiotherapy and chemotherapy function through the induction of cytoplasmic DNA, thus upregulating its pro-inflammatory sensing pathways. New drugs are emerging that target elements in various downstream pathways, and their efficiency in lung carcinoma warrants further clinical trials. Besides enhancing the production of IFN and cytokines, STING agonist also induces tumor cell apoptosis. Both contribute to the recruitment of adaptive immune cells like T lymphocytes. STING agonist can also help maintain CD8^+^ T-cells and potentiate their differentiation, facilitating the anti-tumor effect of CAR-T-cell therapy. The simultaneous induction of PD-L1 indicates that STING agonist can be combined with ICB (immune checkpoint blockade) therapy. More potential combination drug therapies remain to be explored.

The current cancer therapies exploiting cytoplasmic DNA are mostly based on promoting the inflammatory responses in early and chromosomally stable tumors by activating cytoplasmic sensing pathways (**Figure 4**). Nevertheless, if tumor cells have already taken advantage of cytoplasmic sensing pathways to suppress anti-tumor functions, hyperactivation of these pathways may inadvertently worsen the state of illnesses. The specific tumor stage, CIN state, genotype, and basal level of cGAS-STING activation will likely determine the therapeutic responses of the host to STING agonists or antagonists. Therefore, a better understanding of the careful selection of patients is needed to resolve which patients will benefit from pharmacologic therapy that activates or inhibits these important pathways (19, 63, 152).

#### 6.2.1 Common therapies for cancer

Commonly potent cancer strategies are radiotherapy and chemotherapy, such as cisplatin, etoposide, and topotecan, which assist in generating cytoplasmic DNA and invoke STING-dependent IFN production for anti-tumor immunity (190). Conversely, patients with STING expression defects may have resistance to these therapies in certain types of cancer. Moreover, the mechanism of radiation-induced immune stimulation is dose-dependent. When the radiation is delivered at a high dose, induced Trex1 degrades the accumulated cytoplasmic DNA in irradiated tumor cells, which precludes the activation of cGAS-STING-IFN signaling and dampens immune responses. For comparison, tumor cells can be optimally stimulated to produce IFN and prime tumor-specific CD8^+^ T-cells at a low dose given below the threshold of Trex1 induction (147).

Poly (ADP-ribose) polymerase (PARP) is a key DNA repair enzyme, and its inhibitors (PARPi) function as drugs to enhance the sensitivity of radiotherapy and chemotherapy *via* promoting more cytoplasmic DNA production (191, 192). PARPi has been verified to elicit cGAS-STING signaling in both tumor and immune cells to increase immune infiltration and pro-inflammatory signaling (193). By now, PARPi has been approved for treating ovarian and breast cancer with mutations in *BRCA1* and *BRCA2*, two major regulator genes of homologous recombination repair. This narrow therapeutic scope should be expanded as PARPi can potentially treat other cancer types like lung cancer (194, 195). Additionally, the particular stimulus of DNA damage may elicit disparate pathways to activate STING signaling. For instance, cytoplasmic DNA produced by etoposide can trigger STING-mediated secretion of IFN in a cGAS-independent manner (196).

STING agonists are verified to induce tumor cells apoptosis, allowing the leak of antigen-presenting molecules such as TAP1, TAP2, and MHC-I *via* the upregulation of IFN to further cross-prime anti-tumor T-cells and enhance immunosurveillance of tumors (63, 146, 152, 197). Since the cGAS-STING pathway can promote the maintenance of stem-like central memory CD8^+^ T-cells and augment their differentiation by regulating the expression of transcription factor TCF1 and restricting Akt activity, respectively, STING agonists are suggested to potentiate anti-tumor responses of CAR-T-cell therapies through enhancing the production of stem-like central memory CD8^+^ T-cells (198). Moreover, tumor cells treated with STING agonists markedly increase PD-L1 expression and pro-inflammatory cytokines, indicating that STING agonists are ideal partners of ICB therapies (138, 152, 199). For more information, findings using mouse models show that low-dose injection of STING agonists appears to produce effective anti-tumor-associated T-cell responses. At the same time, high repeated doses may impair T-cell responses and the formation of immune memory (152). Thus, low STING agonists may be more helpful as they are sufficient to generate adaptive immune responses. Systemic treatment with STING agonists has been verified to exhibit less efficacy than intertumoral delivery, partly due to the production of B cell-derived IL-35 that hinders NK cell-mediated responses. Specific knockdown of IL-35 in B cells or the usage of IL-35 blocking antibodies in combination with STING agonists can maximize anti-tumor effects in a variety of tumor models (155). This represents a novel strategy of STING agonists for controlling tumor progression.

#### 6.2.2 Therapies for specific types of lung cancer

Currently, many therapies for NSCLC have been created with different clinical responses. Cytoplasmic sensing pathways, especially STING-related ones, play a vital role in enhancing or inhibiting the effects of these therapies.

Recent research about lung cancer showed that low-dose carboplatin, the cornerstone of platinum drugs in lung cancer, changes the ‘cold’ tumor with less anti-tumor cell infiltration into a ‘hot’ tumor with higher infiltration *via* the signaling center STING and increases PD-L1 expression (200, 201). High densities of adaptive immune cells like CD3^+^ or CD8^+^ T-cells represent favorable prognoses for patients (138, 152). In addition, cisplatin also increases the activation of the STING pathway and PD-L1 expression in LSCC and LUAD preclinical models (19, 202). Therefore, combining these chemotherapies and ICB therapies like anti-PD-1/PD-L1 can potentiate the therapeutic effect, and it is a standard therapeutic method for NSCLC patients (19, 202).

Though ICB therapies show potent treatment effects, some patients have failed to benefit from them, including those with KL tumors (168). Designing methods to derepress STING in KL tumors can make them sensitive to ICB (163). Unfortunately, emerging strategies like utilizing STING agonists to activate the STING pathways are less effective in KL tumors. However, they could be resultful in combination with epigenetic methods that increase STING levels. As mentioned above, hyperactivation of DNMT1 and EZH2 activity is a factor in the decreased STING level in LKB1 loss tumors. As a result, treatment with the combination of STING agonists and epigenetic methods like DNMT1 and EZH2 inhibitors could restore STING and cure patients with KL tumors (163).

## 7 Concluding remarks and future directions

Cytoplasmic DNA, both endogenous and exogenous, act as a danger signal to initiate the innate immune response in metazoan cells. Innate immunity is the first line against viral infection, and its conservative pattern maintains its resistance against viral revolution and variance. Nevertheless, given the universality of cytoplasmic DNA sensing, the sterile immune response triggered by self-derived DNA has been well documented to play a vital role in autoimmune diseases and chronic diseases, especially in cancer. Downstream of cytoplasmic DNA sensors, including IFN induction, cytokine production, and cell death, are basic components of innate immune responses that form anti-viral status to prevent viral replication and spread and eliminate damaged components in the organism. Sustained activation of the inflammatory response caused by prolonged sensing of cytoplasmic DNA fragments, which derive from either the counteraction strategy of pathogens or sterile damage devoid of infection, always leads to inflammatory cell death, cytokine storm, and tissue damage, as well as mortality. It is especially the case in many pulmonary inflammatory diseases, where the self-DNA release was discovered, and relative sensing pathways were associated with the pathogenesis.

The crosstalk between different cytoplasmic DNA sensors and their downstream signaling pathways is complicated and shows distinct cell-type-dependent patterns. Though the IFN response is the most typical and recognized downstream of mammalian STING, more and more recent studies have established the critical role of IFN-independent STING signaling in anti-viral response and pathology of lung inflammation and tumor (55, 203). It is the case for SAVI, in which mutant STING triggers inflammatory autoimmune disease through activating STAT1 in T-cells (66). STING activation can also lead to NLRP3-mediated pro-inflammatory cytokine production and secretion in emphysema and IPF (16, 91). During SARS-CoV-2 infection, the vascular endothelial cells and lung epithelial cells exhibit cGAS-STING activation in response to damaged DNA fragments, while the downstream signaling of immune response may be modulated to restrict IRF3 transcription by the evasion strategy of the invading pathogens (23, 110). As cardinal cells of innate immunity, infected macrophages and monocytes also allow the involvement of inflammasomes and the subsequent inflammatory cell death. However, the specific mechanism to activate those inflammasomes in response to various viruses is unclear (21). The ability of AIM2 and NLRP3 to recognize DNA may account for their upregulation in patients with severe COVID-19 (21, 22). In contrast, why and how lung epithelial cells resist inflammasome activation in response to SARS-CoV-2 infection remains to be further explored. Therefore, investigating the heterogeneous functions of each pathway in different innate immune cells and how they contribute to the ongoing inflammation in lung tissues is of great clinical value.

Cytoplasmic DNA and its sensing pathways consolidate the salient status of innate immunity in lung inflammation. Targeting innate immunity is always a focus of lung disease interventions. Though preclinical results targeting cytoplasmic DNA-related pathways are promising, many obstacles remain to be solved. Several therapies, such as JAK inhibitors for SAVI, are limited to the milder phenotype, suggesting the importance of early diagnoses and intervention (97). The pathological heterogeneity of COVID-19 patients, including temporal patterns of IFNs and cytokines, severity, and age, remains challenging for treating SARS-CoV-2 infection. Therapies always have to pay more attention to the balance between anti-viral responses and pathological inflammation. The novel progresses in investigating innate sensing in lymphoid cells, such as T and B lymphocytes and NK cells (55, 70, 155, 204), have thrown light on the potent roles of cytoplasmic DNA in adaptive immunity and lead to development of combinatorial therapeutic strategy.

Further studies are warranted to investigate how cytoplasmic DNA and its signaling components potentiate the adaptive cell-mediated anti- or pro-inflammation and tumor effects in lung pathology, giving insight into the development of immunotherapies like CAR-T. Currently, few studies focus on the role of cytoplasmic DNA sensing pathways in LSCC and small cell lung cancer. Therefore, we only clarify the roles in LUAD rather than distinguishing specific lung cancer subtypes. Future research is recommended to make up this vacancy. Additionally, it is intriguing that the aging process, which gives rise to the accumulation of endogenous cytoplasmic DNA, includes sterile inflammation as one of its hallmarks (15, 29). This indicates that cytoplasmic DNA may also be a potential anti-senescence target in treating aging-related chronic inflammatory diseases and extending the human lifespan.

## Author contributions

JL designed, supervised and supported the whole project. JL, SM and FN revised the manuscript. HX, YS and JL designed the study and wrote the manuscript. All authors contributed to the article and approved the submitted version.
